# Illicit Drug Use Is a Significant Risk Factor for Loss to Follow Up in Patients with HIV-1 Infection at a Large Urban HIV Clinic in Tokyo

**DOI:** 10.1371/journal.pone.0072310

**Published:** 2013-08-07

**Authors:** Takeshi Nishijima, Hiroyuki Gatanaga, Hirokazu Komatsu, Misao Takano, Miwa Ogane, Kazuko Ikeda, Shinichi Oka

**Affiliations:** 1 AIDS Clinical Center, National Center for Global Health and Medicine, Tokyo, Japan; 2 Center for AIDS Research, Kumamoto University, Kumamoto, Japan; 3 Department of Community Care, Saku Central Hospital, Nagano, Japan; University of Athens, Medical School, Greece

## Abstract

**Background:**

Loss to follow up (LTFU) is an important prognostic factor in patients with HIV-1 infection. The impact of illicit drug use on LTFU of patients with HIV-1 infection is unknown in Japan.

**Methods:**

A single center observational study was conducted to elucidate the impact of illicit drug use on LTFU at a large HIV clinic in Tokyo. LTFU was defined as those who discontinued their visits to the clinic for at least 12 months and were not known to be under the care of other facilities or have died within 12 months of their last visit. Patients who first visited the clinic between January 2005 and August 2010 were enrolled. Information on illicit drug use was collected in a structured interview and medical charts. Comparison of the effects of illicit drug use and no use on LTFU was conducted by uni- and multi-variate Cox hazards models as the primary exposure.

**Results:**

The study subjects were 1,208 patients, mostly Japanese men, of relatively young age, and infected through homosexual contact. A total of 111 patients (9.2%) were LTFU (incidence: 24.9 per 1,000 person-years). Among illicit drug users and non users, 55 (13.3%) and 56 (7.1%) patients, respectively, were LTFU, with incidence of 35.7 and 19.2 per 1,000 person-years, respectively. Uni- and multi-variate analyses showed that illicit drug use was a significant risk for LTFU (HR=1.860; 95% CI, 1.282-2.699; p=0.001) (adjusted HR=1.544; 95% CI, 1.028-2.318; p=0.036). Multivariate analysis also identified young age, high CD4 count, no antiretroviral therapy, and no health insurance as risk factors for LTFU.

**Conclusions:**

The incidence of LTFU among illicit drug users was almost twice higher than that among non users. Effective intervention for illicit drug use in this population is warranted to ensure proper treatment and prevent the spread of HIV.

## Introduction

The introduction of highly-active antiretroviral therapy (HAART) has markedly improved the prognosis of patients with HIV-1 infection [1,2]. Patients with HIV-1 infection need to maintain a good level of adherence to antiretroviral therapy (ART) and frequent visits to the health facilities for monitoring treatment efficacy and safety, with regard to the suppression of HIV-1 viral load, recovery of immune function, and improvement of prognosis and survival [3,4]. Those who discontinue medical follow up are likely to develop AIDS-defining illness and die, compared to those who continue their visits [5,6]. Thus, loss to follow up (LTFU) influences prognosis of patients with HIV-1 infection [7–11].

Among patients with HIV-1 infection, those who use illicit drugs are associated with lower ART uptake and inferior adherence to treatment [12–15], which lead to suboptimal treatment outcome, compared with patients with other risk categories [16–18]. However, illicit drug users are one of the “difficult to reach” populations and it is difficult to obtain accurate data on them [19]. It is especially difficult in Japan to collect data on illicit drug users, because of a strong government policy against illicit drug use and extremely low lifetime prevalence of illicit drug use in the general population (2.9% in 2009 according to the Nationwide General Population Survey on Drug Use and Abuse) [20,21] (http://www.ncnp.go.jp/nimh/pdf/h21.pdf. in Japanese) (http://www.mhlw.go.jp/bunya/iyakuhin/yakubuturanyou/torikumi/dl/index-04.pdf. in Japanese). Thus, there are no data on illicit drug use among patients with HIV-1 infection, and the impact of such use on prognosis of HIV-1 infected patients in Japan [20,22].

Based on the abovementioned background, the aim of the present study was to elucidate the impact of illicit drug use on LTFU among patients with HIV-1 infection at a large urban HIV clinic in Tokyo, Japan.

## Methods

### Ethics Statement

This study was approved by the Human Research Ethics Committee of the National Center for Global Health and Medicine, Tokyo, Japan. The Committee waived a written informed consent, since this study only uses data of anonymized patients obtained from a routine practice. The study was conducted according to the principles expressed in the Declaration of Helsinki.

### Study design

This study was designed and reported according to the recommendations of STROBE (Strengthening the Reporting of Observational studies in Epidemiology) statement [23]. We performed a single center observational study of patients with HIV-1 infection to elucidate whether illicit drug use is a risk factor for LTFU in a large urban HIV clinic in Tokyo. The AIDS Clinical Center is one of the largest clinics for HIV care in Japan with more than 3,300 registered patients. Considering that the total reported number of patients with HIV-1 infection is 21,415 by the end of 2011, this clinic treats approximately 15% of the HIV-1 infected patients in Japan (http://api-net.jfap.or.jp/status/2011/11nenpo/hyo_02.pdf. in Japanese).

### Study subjects

The study population was patients with HIV-1 infection, aged >17 years, who visited our clinic for the first time from January 1, 2005 to August 31, 2010. The exclusion criteria were; 1) those who came for the second opinion and 2) those who were referred to other facilities on their first or second visit. They were excluded because the structured interview on social demographics was often not conducted for these patients. Patients who refused to have their data included in the study were also excluded. Patients were followed up until December 31, 2012.

### Measurements

Variables were collected through a structured interview conducted at the first visit of each patient as part of routine clinical practice by the nurses specializing at the HIV outpatient care. The interview by these “coordinator nurses” included the following variables: history of illicit drug use and injection drug use (and type of illicit drugs if available), health insurance status, perceived route of transmission, sexuality, and whether living alone or with someone.

Because the interview could underestimate the prevalence of illicit drug use, we also searched the medical records for information on illicit drug use and related variables covering the period from the first visit to December 2012. Information on age, sex, ethnicity, treatment status for HIV infection, and history of AIDS [(defined as history of or concurrent 23 AIDS-defining diseases set by the Japanese Ministry of Health, Labour and Welfare) (http://www.haart-support.jp/pdf/guideline2012.pdf. in Japanese)] were extracted from the medical records. The laboratory data of CD4 cell count, HIV-1 viral load, and hepatitis C antibody on the first visit were also collected, and if these test results were not available on that day, the data within three months from the first visit were used.

### Definition of loss to follow up

LTFU was defined according to the literature as follows: patients who discontinued their visits to the AIDS Clinical Center for at least 12 months after the last visit and who were not known to be under the care of other medical facilities or have died within 12 months of their last visit [24]. At our clinic, all patients provide their phone numbers at the first visit, and when they miss the scheduled visit, the abovementioned “coordinator nurse” calls the patient to make another appointment, or leave a message to visit if the patient does not answer the phone. If the patient does not visit the clinic after the first call, the nurses continue calling the patient every three months up to one year. For the majority of lost cases, we checked whether the patient went to seek care in another hospital, because in Japan only a few clinics provide HIV care, due to the low prevalence of HIV-1 infection (0.016%) (http://www.stat.go.jp/english/data/kokusei/pdf/20111026.pdf) (http://api-net.jfap.or.jp/status/2011/11nenpo/hyo_02.pdf. in Japanese). Thus, even if a patient stopped visiting our clinic and started seeking help at other facilities without informing the first health care provider, the new facility almost always contacts the original facility to obtain medical information.

### Statistical analysis

Patients’ characteristics and social demographics were compared between those who were LTFU and those who continued visiting the clinic by the Student’s *t*-test for continuous variables and by either the χ^2^ test or Fisher’s exact test for categorical variables.

The time to LTFU as defined above was calculated from the date of the first visit to the date of LTFU. Censored cases represented those who were referred to other facilities, or who died within 12 months of their last visit, or at the end of follow-up period. The time from the first visit to LTFU was analyzed by the Kaplan Meier method for patients who experienced illicit drug use and those who did not, and the log-rank test was used to determine the statistical significance. The Cox proportional hazards regression analysis was used to estimate the impact of illicit drug use over non users on the incidence of LTFU as a primary exposure. The impact of each basic demographics, baseline laboratory data, and other medical conditions listed above was also estimated with univariate Cox proportional hazards regression.

To estimate the unbiased prognostic impact of illicit drug use over non-users for LTFU, we conducted three models using multivariate Cox proportional hazards regression analysis. Model 1 was the aforementioned univariate analysis for illicit drug use over non users. Model 2 included basic demographics (age and Japanese) plus model 1. In model 3, we added CD4 count, ART, and health insurance status, because they showed significant relationship with LTFU in univariate analysis and the literatures showed a high CD4 count, without ART and without health insurance is a risk factor for LTFU [11,24,25]. History of AIDS and HIV-1 viral load were not added to the model, based on their multicollinearity with CD4 count and ART, respectively.

To elucidate whether the impact of illicit drug use on LTFU is affected by sexual behavior, we divided patients into MSM and non-MSM groups. Then, the abovementioned multivariate analysis was conducted for each group.

Statistical significance was defined at two-sided *p* values <0.05. We used hazard ratios (HRs) and 95% confidence intervals (95% CIs) to estimate the impact of each variable on LTFU. All statistical analyses were performed with The Statistical Package for Social Sciences ver. 20.0 (SPSS, Chicago, IL).

## Results

**Figure 1:**
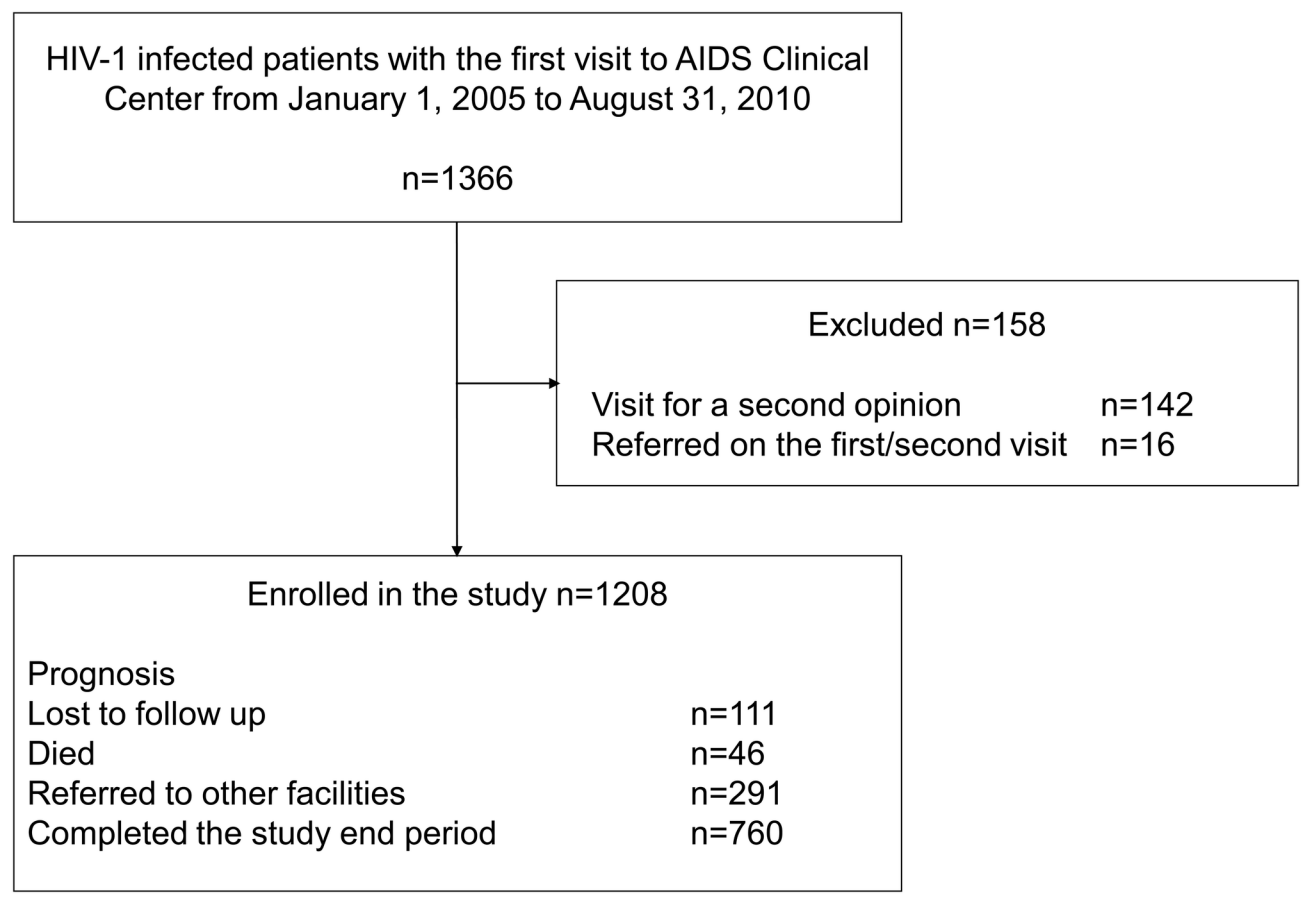
Patient enrollment process.

A total of 1,366 patients with HIV-1 infection visited the AIDS Clinical Center for the first time during the study period. 142 patients visited for a second opinion and 16 patients were referred to other facilities on their first or second visit. Thus, 158 patients were excluded from the analysis (Figure 1). Table 1 summarizes characteristics of the 1,208 patients included in this study. The perceived route of transmission was homosexual contact in 948 (79%), heterosexual contact in 173 (14%), injection drug use in 22 (2%), contaminated blood product in 11 (1%), vertical transmission in 1 (0.1%), and unknown in 53 (4%). Further analysis indicated that 973 (81%) patients were MSM regardless of the perceived route of transmission (e.g., if a patient considered that they were infected with HIV-1 through injection drug use and they were MSM, they were classified to MSM in this study). The study patients were mostly Japanese men of relatively young age (mean: 36 years). Most patients were ART-naïve, with a median CD4 count of 245/µl.

**Table 1:** Baseline demographics and laboratory data for all study population, those who were lost to follow up and those who continued the visits.

	All (n=1,208)	Lost follow up (n=111)	Others (n=1,097)	P value
Sex (male), n (%)	1125 (93)	103 (93)	1022 (93)	0.84
Median (IQR) age	36 (29-43)	31 (25-39)	36 (30-43)	<0.01
Illicit drug use, n (%)	415 (34)	55 (50)	360 (33)	<0.01
Injection drug use, n (%)	53 (4)	8 (7)	45 (4)	0.14
Methamphetamine use, n (%)	63 (5)	10 (9)	53 (5)	0.07
Arrested due to illicit drug, n (%)	27 (2)	5 (5)	22 (2)	0.09
Median (IQR) CD4 count (/µl)^a^	245 (101-380)	391 (313-515)	231 (84-359)	<0.01
Median (IQR) HIV-1 viral load (log_10_/ml)^b^	4.59 (3.89-5.18)	4.32 (3.80-4.75)	4.64 (3.91-5.20)	0.03
AIDS, n (%)	323 (27)	10 (9)	313 (29)	<0.01
On antiretroviral therapy, n (%)	131 (11)	5 (5)	126 (12)	0.02
Positive HCV antibody, n (%)	46 (4)	2 (2)	44 (4)	0.43
Men who have sex with men, n (%)	973 (81%)	89 (80)	884 (81)	0.90
Transmission category, n (%)				0.51
Homosexual contact	948 (79)	84 (76)	864 (79)	
Heterosexual contact	173 (14)	19 (17)	154 (14)	
Injection drug use	22 (2)	4 (4)	18 (2)	
Contaminated blood product	11 (1)	0	11 (1)	
Vertical transmission	1 (0.1)	0	1 (0.1)	
Unknown	53 (4)	4 (4)	49 (5)	
Ethnicity, n (%)^c^				0.02
Japanese	1070 (89)	92 (83)	978 (89)	
Asian	70 (6)	7 (6)	63 (6)	
White	27 (2)	2 (2)	25 (2)	
Black	26 (2)	7 (6)	19 (2)	
Latino	12 (1)	2 (2)	10 (0.9)	
Health insurance status, n (%)				<0.01
Without insurance	55 (5)	13 (12)	42 (4)	
With insurance/public assistance	1153 (95)	98 (88)	1055 (96)	
Working status, n (%)^d^				0.09
Unemployed	230 (19)	23 (21)	207 (19)	
With any job	909 (75)	77 (69)	832 (76)	
Student/housewife	68 (6)	11 (10)	57 (5)	
Living alone, n (%)^e^	532 (44)	46 (41)	486 (44)	0.62
Median (IQR) follow up days	1384.5 (732-1991)	266 (58-800)	1454 (914-2053)	<0.01

Data for ^a^ two, ^b^ four, ^c^ three, ^d^ one, and ^e^ fifteen cases, respectively, are missing

Based on the interview and medical records, 34% of the patients were illicit drug users (including injection drug users), 4% were injection drug users and 5% had used methamphetamine. Of the total, 2% were detained or arrested for possession or use of illicit drugs. Among illicit drugs, amyl nitrite and 5-methoxy-diisopropyltryptamine were the most commonly named by study patients (amyl nitrite and 5-methoxy-diisopropyltryptamine became prohibited substance by law in 2006 and 2005, respectively, in Japan) [26]. Methamphetamine, 3,4-methylenedioxymethamphetamine, cannabis, heroin, cocaine, and opium were also mentioned (numbers not counted except for methamphetamine).

LTFU patients were significantly more likely to be illicit drug users and tended to use methamphetamine and be arrested/detained due to illicit drug use than those who continued to visit the clinic. LTFU tended to be non-Japanese, younger age, had higher CD4 count, and less likely to have a history of AIDS, on ART, and covered by health insurance/public assistance, compared to the patients who continued to visit the clinic (Table 1).

**Figure 2:**
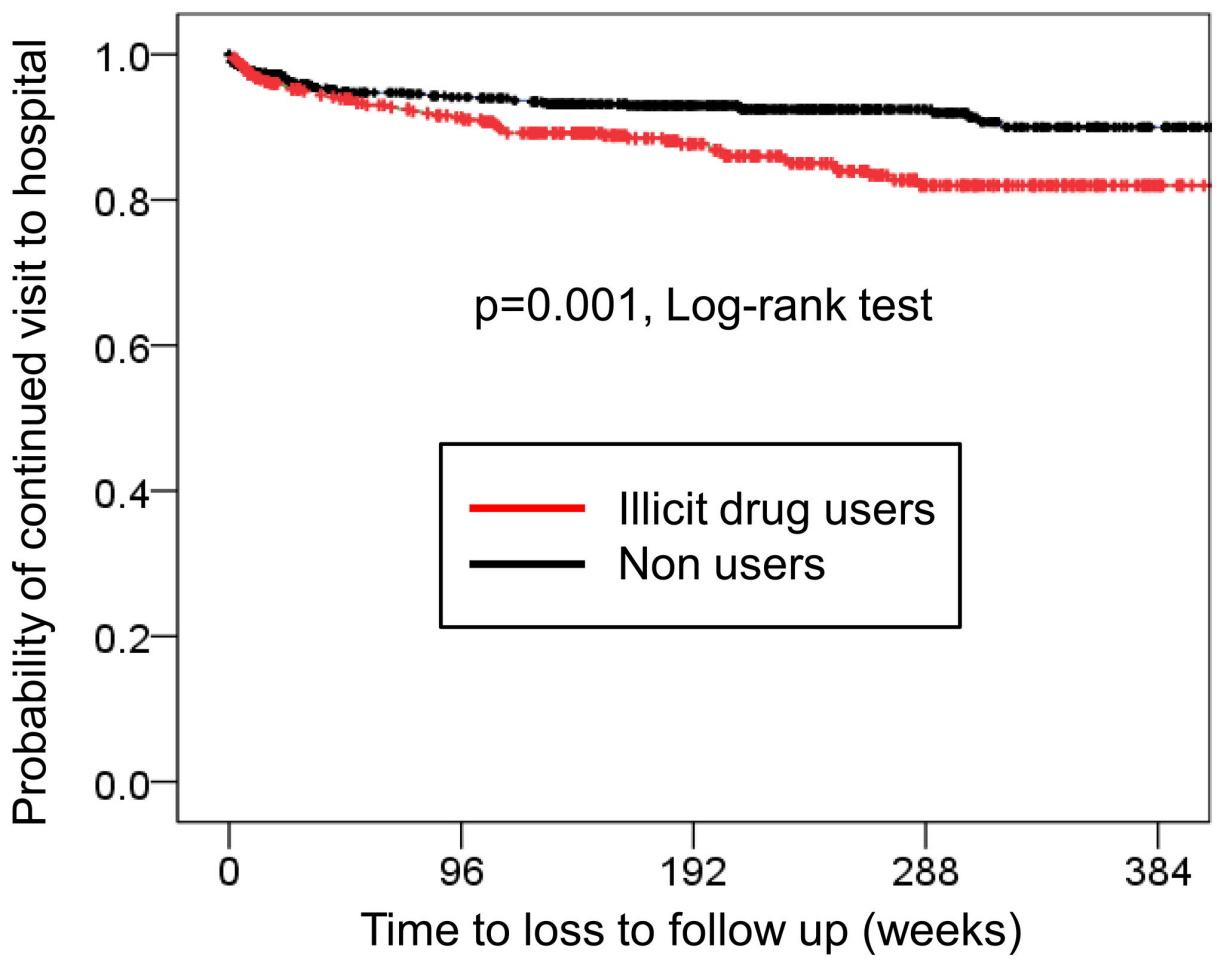
Kaplan-Meier curve showing time to loss to follow up for illicit drug users and non users. Compared to non drug users, illicit drug users were more likely to discontinue their visits to the hospital (p=0.001, Log-rank test).

Among the 1,208 patients included in the study, 111 (9.2%) were LTFU as defined above, with an incidence of 24.9 per 1,000 person-years. The median time from the first visit to LTFU was 266 days (IQR 58-800 days). Among illicit drug users (n=415) and non-users (n=793), 55 (13.3%) and 56 (7.1%) patients, respectively, were LTFU, with incidence of 35.7 and 19.2 per 1,000 person-years, respectively. Figure 2 shows the time from the first visit to LTFU by the Kaplan Meier method for the two groups. Illicit drug users were significantly more likely to stop visiting the clinic, compared to non-users (p=0.001, Log-rank test). The total observation period was 1,541.4 patient-years [median, 1,405 days, interquartile range (IQR), 674-2,029 days] for illicit drug users and 2,920.4 patient-years (median, 1,371 days, IQR, 759-1943 days) for non users.

Univariate analysis showed a significant relationship between illicit drug use and LTFU (HR=1.860; 95% CI, 1.282-2.699; p=0.001) (Table 2). Furthermore, young age, high baseline CD4 count, low HIV viral load, no history of AIDS, non Japanese, no ART, and no health insurance/public assistance were associated with LTFU. Injection drug use and methamphetamine use, respectively, were marginally associated with LTFU (injection drug use: HR=1.808; 95% CI, 0.880-3.713; p=0.107) (methamphetamine use: HR=1.684; 95% CI, 0.879-3.225; p=0.116).

**Table 2:** Univariate analysis to estimate the risk of various factors for loss to follow up.

	Hazard ratio	95% CI	P value
Illicit drug use	1.860	1.282-2.699	0.001
Injection drug use	1.808	0.880-3.713	0.107
Methamphetamine use	1.684	0.879-3.225	0.116
Arrested/detained due to illicit drug	1.981	0.808-4.859	0.135
Male gender	0.961	0.468-1.974	0.961
Men who have sex with men	0.926	0.581-1.477	0.747
Age ≤30 years	Reference		
30 < Age ≤40 years	0.455	0.299-0.692	<0.001
Age >40 years	0.320	0.190-0.538	<0.001
CD4 count ≤200/µl	Reference		
200 < CD4 count ≤350/µl	2.536	1.318-4.878	0.005
CD4 count >350/µl	7.651	4.309-13.59	<0.001
HIV-1 viral load per log_10_/ml	0.846	0.730-0.981	0.027
History of AIDS	0.269	0.140-0.514	<0.001
Positive HCV antibody	0.466	0.115-1.888	0.285
Japanese	0.559	0.337-0.926	0.024
On antiretroviral therapy	0.402	0.164-0.986	0.046
With any job	0.870	0.549-1.376	0.551
On health insurance/public assistance	0.249	0.139-0.444	<0.001
Living alone	0.949	0.649-1.388	0.788

Multivariate analysis identified illicit drug use as a significant risk for LTFU after adjustment for age and Japanese (adjusted HR=1.802; 95% CI, 1.209-2.686; p=0.004) (Table 3, Model 2), and also after adjustment for other risk factors (adjusted HR=1.544; 95% CI, 1.028-2.318; p=0.036) (Table 3, Model 3). Young age, high baseline CD4 count, no ART, and no health insurance/public assistance also persisted to be risk for LTFU in multivariate analysis.

**Table 3:** Multivariate analysis to estimate the risk of illicit drug use for loss to follow up.

	Model 1 Crude (n=1,208)		Model 2 Adjusted (n=1,208)		Model 3 Adjusted (n=1,206)	
	HR	95% CI	Adjusted HR	95% CI	Adjusted HR	95% CI
Illicit drug use^†^	1.860	1.282-2.699	1.770	1.208-2.592	1.513	1.018-2.248
Age ≤30 years^†^			Reference		Reference	
30< Age ≤40 years^†^			0.462	0.304-0.703	0.467	0.303-0.720
Age >40 years^†^			0.360	0.212-0.609	0.442	0.259-0.752
Japanese			0.472	0.286-0.779	0.798	0.443-1.436
CD4 count ≤200/µl^†^					Reference	
200< CD4 count ≤350 /µl^†^					2.221	1.148-4.297
CD4 count >350/µl^†^					7.087	3.951-12.71
On antiretroviral therapy^†^					0.366	0.147-0.912
With health insurance/public assistance^†^					0.204	0.102-0.409

†

p<0.05 in Model 3

Subgroup analysis of the patients stratified by sexual behavior showed that among MSM patients (n=973), the impact of illicit drug use on LTFU was slightly more evident (adjusted HR=1.641; 95% CI, 1.061-2.538; p=0.026) (Table 4) than in the total population (adjusted HR=1.544; 95% CI, 1.028-2.318; p=0.036) (Table 3, Model 3). On the other hand, illicit drug use had no significant impact in non-MSM patients (n=233) (adjusted HR=1.119; 95% CI, 0.248-5.053; p=0.883).

**Table 4:** Multivariate analysis to estimate the risk of illicit drug use for loss to follow up stratified by sexual behavior.

	Adjusted HR	95% CI	P value
MSM (n=973)	1.641	1.061-2.538	0.026
Non MSM (n=233)	1.119	0.248-5.053	0.883

Adjusted by variables in Table 3, Model 3 (age, Japanese, CD4 count, antiretroviral therapy, and health insurance) MSM: men who have sex with men

## Discussion

At this large urban HIV clinic in Tokyo, 9.2% of the patients were lost to follow up, with an incidence of 24.9 per 1,000 person-years. Furthermore, 34% of the study patients were illicit drug users and the incidence of LTFU for illicit drug users was almost twice higher than that for non users (35.7 and 19.2 per 1,000 person-years, respectively). Illicit drug use was identified as a significant risk for LTFU in uni- and multi-variate analyses (HR=1.860; 95%CI, 1.282-2.699; p=0.001) (adjusted HR=1.544; 95% CI, 1.028-2.318; p=0.036). The impact of illicit drug use on LTFU was slightly more evident among MSM than in the total study population.

To our knowledge, only a few studies have examined the impact of non-injection illicit drug use on LTFU [9,27], and this is the first such study conducted in Asia. The results showed that illicit drug use is a risk factor for LTFU, which is a marker for prognosis in patients with HIV-1 infection [7–11]. The result emphasizes the need for effective prevention and intervention strategies for illicit drug use in patients with HIV-1 infection in Japan. The finding of a more evident impact of illicit drug use in MSM patients also highlights the need for close monitoring of adherence to HIV care in this group of patients.

Among patients with HIV-1 infection, the prognosis of injection drug users is reported to be worse than that of non-injection drug users [28]. However, this study primarily focused on illicit drug use as a whole, rather than injection drug use for two main reasons; First, only a few studies focused on illicit drug use among HIV-1 infected patients, although a large number of studies focused on injection drugs [24,25,27,29,30]. Illicit drug use in patients with HIV-1 infection is an important issue, because not only illicit drug use lead to inferior treatment outcome compared with non users [16–18], but also non injection drug users are prone to practice high risk sexual behaviors, which might lead to transmission of HIV and other infectious diseases [14,31]. Furthermore, illicit drug use, especially opioid use, can be a trajectory into injection drug use [32,33]. Second, because only 0.5% of the patients were infected with HIV-1 through injection drug use by the end of 2011 in Japan (according to a nationwide surveillance conducted by the AIDS Surveillance Committee of the Ministry of Health, Labour and Welfare that covered all reported cases with HIV-1 infection), the anticipated prevalence of injection drug use was very low (http://api-net.jfap.or.jp/status/2011/11nenpo/hyo_02.pdf. in Japanese). Surprisingly, the prevalence of injection drug use was 4% in this study, the number is much higher than what the AIDS Surveillance Committee reported. This suggests a substantial underreporting for injection drug use as a route of transmission from the patients.

In the planning and design of effective prevention and intervention strategies for illicit drug users with HIV-1 infection in Japan, the unique circumstances related to this issue need to be taken into consideration. First, on one hand, the government maintains a strict punitive policy against illicit drug use and this policy has been one of the factors that helped maintain a relatively low prevalence of illicit drug use (lifetime prevalence 2.9%) [21] (http://www.ncnp.go.jp/nimh/pdf/h21.pdf. in Japanese). On the other hand, possibly due in part to severe criminalization of drug use, treatment and rehabilitation schemes for drug users remain poorly developed [20,34].

Second, most injected drugs in Japan are methamphetamine: In 2010, the number of arrested illicit drug users categorized by each drug was the largest for methamphetamine (12,200), while the numbers for other injectable drugs, such as heroin and cocaine were very small (22 and 112, respectively) (http://www.mhlw.go.jp/bunya/iyakuhin/yakubuturanyou/torikumi/dl/index-01.pdf. in Japanese). In the study patients, injection drug users and methamphetamine users also appeared to overlap considerably. Evidence from other countries shows that methamphetamine use has gained popularity among MSM, and methamphetamine use is strongly associated with high-risk sexual behavior [35–38]. Thus, any intervention for injection drug users with HIV-1 infection in Japan needs to take into consideration the frequent use of methamphetamines.

Several limitations need to be acknowledged. First, due to the nature of single-center study, the results of this study do not necessarily represent all patients with HIV-1 infection in Japan. However, as abovementioned, our clinic treats approximately 15% of the total HIV patients in Japan, and furthermore, characteristics of the patients with HIV-1 infection newly diagnosed and reported to the Japanese National HIV Registry in 2011 (n=1529) is very similar to those of the study population: 94% male, 64% infected through homosexual contact, and 59% in their 20s and 30s of age (http://api-net.jfap.or.jp/status/2011/11nenpo/hyo_02.pdf. in Japanese). Most HIV-1 infected patients reside in urban areas such as Tokyo metropolitan area as well. Thus, the discrepancy between the study patients and all HIV patients in Japan should not be too large. Second, the structured interview designed for data collection does not prevent underreporting of illicit drug use. However, underreporting to a certain degree is unavoidable with regard to issues such as illicit drugs [19].

In conclusion, the incidence of LTFU in illicit drug users was almost twice higher than that in non users among patients with HIV-1 infection in Japan. Multivariate analysis identified illicit drug use as a significant risk factor for LTFU, which influences prognosis of patients with HIV-1 infection. Little data is available for illicit drug use in Japan, especially among patients with HIV-1 infection. However, all relevant parties in relation to this issue need to recognize that illicit drug use has spread among patients with HIV-1 infection, and that illicit drugs worsens adherence to HIV care in Japan. Appropriate measures for prevention and intervention of illicit drug use are urgently needed to ensure proper treatment and prevention of spread of HIV infection.
